# The student workforce: untapped possibilities

**DOI:** 10.1111/tct.13217

**Published:** 2020-07-10

**Authors:** Elizabeth S Anderson, Kishan Patel

**Affiliations:** ^1^ Leicester Medical School, George Davies Centre University of Leicester Leicester UK

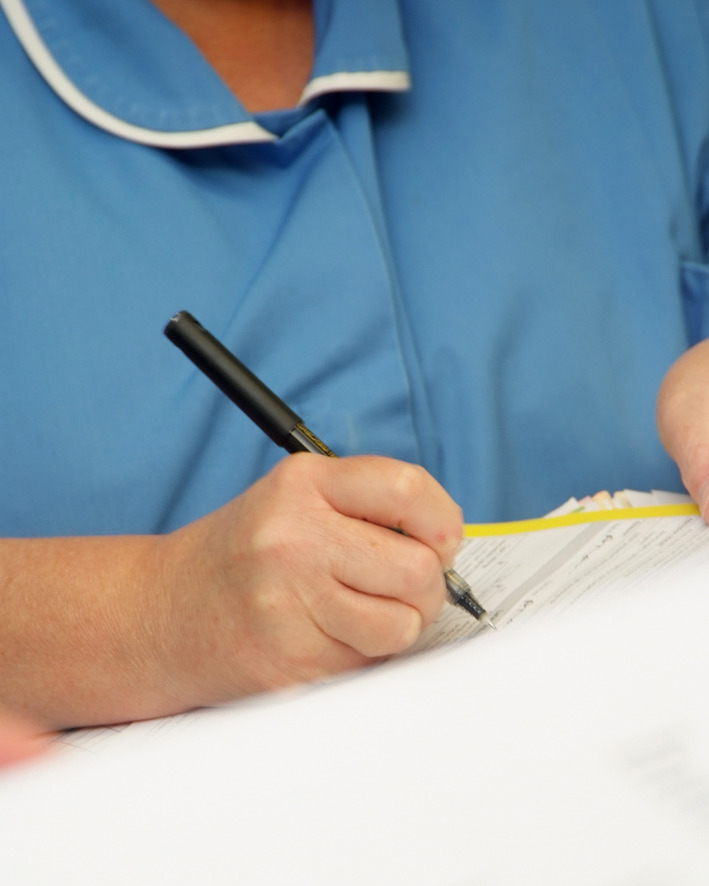

The coronavirus disease 2019 (COVID‐19) pandemic offers us a moment to stop and re‐think what matters in health care professional training. In the United Kingdom (UK) the virus outbreak began on 29 January 2020 and by 13 March 2020 our medical school became virtual. The immediate concern was to expand the health care workforce, with medical and other health care practitioners propelled to the frontline. Medical schools saw large numbers of students wanting, willing and hoping to help. Retired practitioners returned but junior doctors teaching in medical schools were not called, as education must continue. Students, retired practitioners and teaching faculty members want to help and are needed. How can these positive responses be aligned so that caring and learning both continue during emergency situations?

Students’ willingness to help is diminished when they realise that they lack the skill set needed for the frontline in a pandemic. Even newly qualified doctors require a great deal of support as they enter practice. In the UK, medical students spend their earliest years learning about science. In normal times, never mind during a pandemic, they report feeling unprepared for practice in hospitals, despite increased early clinical exposure.^1^ This pattern of an early focus on theory is replicated in other health and social care professional training. As a result, students know little of ward life and interprofessional practice, including handover, how nurses manage wards, doctors’ workloads, systems for recording essential patient data and diagnostic support systems: in effect, how teams work together within systems. In reality, students arrive in clinical practice lost and frozen, like rabbits in headlights, confused about what, where and how?^2^ Since 2010 we have heard urgent calls for medical training to place greater emphasis on team‐based practice, population needs, systems and patient safety.^3^ Although this is not the time to look back with regret, we have failed to modernise training in ways that might have seen students better prepared for the frontline.

… we have failed to modernise training in ways that might have seen students better prepared for the frontline

Are there solutions? Immersion into the realities of practice and teamworking can be found working as a nursing assistant. Some medical schools prepare medical students to be workforce ready at the outset by providing health care nursing assistant training at the start of their curriculum. Gaining these basic skills does not take long.^4^ Our university is conducting a similar trial with 30 medical students gaining the health care nursing assistant certificate in the first semester. These students learned and practised reverse barrier nursing for infection control with senior clinical nurse specialists in week 2 of their health care nursing assistant training! Our evaluation identified that these medical students learned a great deal about team‐based interprofessional practice and gained a deeper respect for nurses and other team members. Conversely, the nurse and specialist trainers reported new learning about the detail of medical training and gained greater insights into doctors’ work patterns and responsibilities. Nursing assistants now form the backbone of any health care system but have little exposure to the details of medical training. Training these medical students to be health care nursing assistants is changing their perceptions of doctors possibly for the first time (paper forthcoming). Our students have gone out to help in the COVID‐19 pandemic armed with their care certificates just 1 year into training. This offers a new route to prepare our workforce and has definitely stopped student laments for ‘more clinical time’ and more ‘patient contact please’.

Meanwhile, 500 retired doctors stepped up within 48 hours in the UK with different challenges, as they are unfamiliar with the new systems and are vulnerable to COVID‐19. Within most medical schools there are junior doctors on Foundation Programmes (training during the first 2 years post‐registration in the UK) taking time out to further careers in research and education who feel ignored.^5^ There are approximately 1000 junior doctors working in their second year and some of these are stepping in for absent clinical‐facing medical educators. They are currently working hard to help students cover lost placement learning. The raw histories and examinations of patients is being replaced with weekly guided e‐learning material using virtual simulation. Students can use ‘choose your own adventure’ simulations, where they navigate a case history by clicking on diagnostic and treatment options, and are taking newly designed histories by video call. Weekly live quizzes summarise the topic. If all of these students carried health care nursing assistant certificates and had the skill set to work in acute or community settings, however, they could be learning at the frontline right now, freeing up our clinical teachers to support them in practice alongside those returning from retirement.

After the COVID‐19 pandemic there will be time to re‐think the early content for health professional education. Training to be a care assistant gives every student vital skills for the workplace and the experience upon which to build later theoretical learning. Learning about the realities of clinical practice at the outset of training delivers a helpful, interprofessional, team‐based and systems‐aware workforce.^4^

Training to be a care assistant gives every student vital skills for the workplace and the experience upon which to build later theoretical learning
